# Asymmetric Fc Engineering for Bispecific Antibodies with Reduced Effector Function

**DOI:** 10.3390/antib6020007

**Published:** 2017-05-16

**Authors:** Eric Escobar-Cabrera, Paula Lario, Jason Baardsnes, Joseph Schrag, Yves Durocher, Surjit Dixit

**Affiliations:** 1 Zymeworks Inc., 540-1385 West 8th Ave, Vancouver, BC V6H 3V9, Canada; plario@gmail.com; 2 Human Health Therapeutics, National Research Council Canada, 6100 Royalmount Ave, Montreal, QC H4P 2R2, Canada; Jason.Baardsnes@cnrc-nrc.gc.ca (J.B.); Joe.Schrag@cnrc-nrc.gc.ca (J.S.); Yves.Durocher@cnrc-nrc.gc.ca (Y.D.)

**Keywords:** Fc engineering, asymmetric bispecific antibody, ion exchange chromatography, knock-out effector function

## Abstract

Asymmetric bispecific antibodies are a rapidly expanding therapeutic antibody class, designed to recognize two different target epitopes concurrently to achieve novel functions not available with normal antibodies. Many therapeutic designs require antibodies with reduced or silenced effector function. Although many solutions have been described in the literature to knockout effector function, to date all of them have involved the use of a specific antibody subtype (e.g., IgG2 or IgG4), or symmetric mutations in the lower hinge or CH2 domain of traditional homodimeric monospecific antibodies. In the context of a heterodimeric Fc, we describe novel asymmetric Fc mutations with reduced or silenced effector function in this article. These heteromultimeric designs contain asymmetric charged mutations in the lower hinge and the CH2 domain of the Fc. Surface plasmon resonance showed that the designed mutations display much reduced binding to all of the Fc gamma receptors and C1q. Ex vivo ADCC and CDC assays showed a consistent reduction in activity. Differential scanning calorimetry showed increased thermal stability for some of the designs. Finally, the asymmetric nature of the introduced charged mutations allowed for separation of homodimeric impurities by ion exchange chromatography, providing, as an added benefit, a purification strategy for the production of bispecific antibodies with reduced or silenced effector function.

## 1. Introduction

Therapeutic antibodies have been developed for the treatment of many disease indications. In some cases, therapeutic efficacy results, at least in part, from the ability of the Fc region of the antibody to mediate one or more effector functions. These effector functions result from the interaction of antibodies and antibody-antigen complexes with cells of the immune system to stimulate a variety of responses, including antibody-dependent cell-mediated cytotoxicity (ADCC) and complement dependent cytotoxicity (CDC) [1,2,3]. Several antibody effector functions are mediated by Fc receptors (FcRs), which bind the Fc region of an antibody. FcRs are defined by their specificity for immunoglobulin isotypes; Fc receptors for IgG antibodies are referred to as FcγR, for IgE antibodies as FcεR, for IgA antibodies as FcαR, and so on. The CDC activity of the antibody is mediated by interactions of the Fc with proteins of the complement system such as C1q. The Fc region of antibodies also mediates functions, such as binding to FcRn, that are independent of antigen binding and confer persistence in circulation and the ability to be transferred across cellular barriers by transcytosis [2].

A number of therapeutic antibody applications require silencing of effector activity such as ADCC or CDC. This may be particularly relevant in the context of bispecific antibodies in which a major class redirects immune cells or engages immunomodulatory targets. For example, one arm of the bispecific antibody could target a tumor associated antigen (TAA), while the other arm may engage receptors on immune cell, such as CD3 on a T-cell. Simultaneous engagement of a T-cell by the Fab arm of the antibody and an NK cell via Fc/FcγR interactions could inadvertently redirect the cytotoxic activity of the NK cell to the T-cell or vice versa. In such circumstances, it may be desirable to minimize or silence effector function mediated by the Fc region of the asymmetric bispecific antibodies.

Recent publications describe strategies that have been used to engineer antibodies with reduced or silenced effector activity [4,5,6,7]. These strategies include reduction of effector function through modification of glycosylation [8,9,10], use of IgG2/IgG4 scaffolds [11,12,13,14], or the introduction of engineered mutations in the hinge or CH2 domain of the Fc region of the antibody [7,13,15,16,17]. Since natural antibodies are homodimeric in nature, so far only symmetric mutations that reduce effector activity have been reported. However, a number of different strategies to produce heterodimeric antibodies have been developed [18,19,20] and are being applied in the design of therapeutic bispecific antibodies. In this work, we made use of a heterodimeric Fc scaffold to design novel asymmetric Fc mutations that can be used in bispecific antibodies that require effector function silencing. We describe the mutations and highlight the other relevant functional characteristics such as stability and purification opportunities resulting from the incorporation of these designs.

## 2. Results

### 2.1. Preparation and Expression of Antibody Constructs (Heteromultimers)

**Figure 1:**
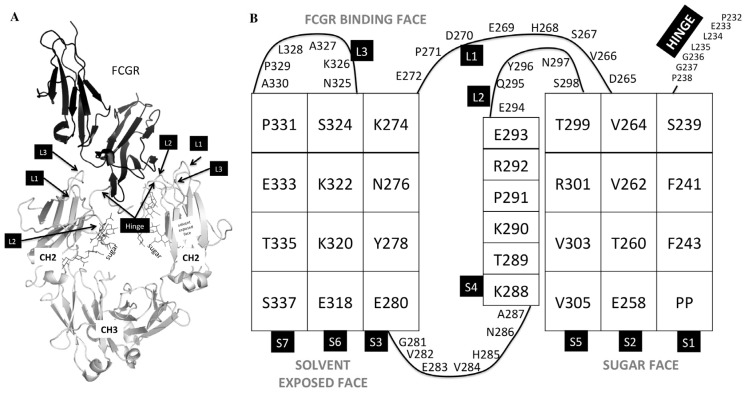
Regions of the Fc region that are involved in FcγR binding. (**A**) 3b/Fc crystal structure (PDB:1E4K) denoting the loops and lower hinge of the CH2 domain of the Fc (shown in gray) that are involved in FcγR binding (shown in black). (**B**) Topology of the CH2 domain. Strands are denoted as S1, S2, S3, S4, S5, S6, and S7; Loops are denoted L1, L2, and L3.

Most antibody constructs described in this work were based on the sequence of the wild-type anti-Her2 antibody trastuzumab CDRs with the IgG1 framework and constant domains. The antibody variants included a common trastuzumab light chain sequence coexpressed with two different heavy chains comprising specific mutations in the CH3 domain that preferentially form a heterodimeric Fc. The mutations used were T350V/L351Y/S400E/F405A/Y407V and T350V/T366L/N390R/K392M/T394W for chains A and B, respectively. Variant 791 showed that these mutations did not change the binding to FcγRs significantly (Table 1). The CH2 mutations contained in the constructs and their location are shown in Table 1 and Figure 1, respectively. Mutations were engineered using a structure guided approach, taking into consideration the structural observation that the two chains of Fc do not interact with the Fc receptors in a symmetric manner. We focused on the L234-G237 region, which since the early 90s was demonstrated to be key for mediating Fc/FcγR interactions [21]. Indeed, variant 1051 (L234A/L235A) is a previously reported knock-out mutation tested in a native homodimer background [4]. Asymmetric antibody construct 1 (AAC1) is an asymmetric version of variant 1051, in which only one of the heavy chains has the L234A/L235A double mutation. AAC2-AAC8 are novel asymmetric designs meant to alter effector activity as well as provide improved developability features. As summarized in Table 1, the mutations had little effect on the expression yield. The majority of the samples showed levels of expression similar to the WT or control.

While the ADCC activity could be tested using an anti-Her2 antibody construct (Section 2.7), the CDC activity of the mutations is evaluated in the context of an anti-CD20 antibody. To that end one of the antibody constructs described here was based on the sequence of the wild-type anti-CD20 antibody rituximab CDRs with the IgG1 framework and constant domains. The bioassay screening for CDC activity is better optimized for antibodies targeting the CD20 receptor than Her2 receptor. As discussed in Section 2.8, we selected AAC5 as the lead design to be cloned into the anti-CD20 antibody rituximab (variant AAC9) for further testing in ADCC and CDC screens. A design with fewer CH3 domain mutations driving the heterodimerization of Fc domain was also introduced (Chain A: T350V/L351Y/F405A/Y407V, and Chain B: T350V/T366L/K392L/T394W) in this system. We have shown in earlier work that these mutations yield pure and stable heterodimeric antibody [19]. The control construct with these heterodimerization mutations and no CH2 mutations is referred to as variant 1261 (Table 2). Similar to what we observed with trastuzumab, all samples expressed well, at levels comparable to the control parent rituximab (not shown).

### 2.2. SPR Binding to Fcγ Receptors

The ability of the asymmetric trastuzumab antibody constructs to bind to FcγRs 2aH, 2aR, 2b, 3aF, 3aV, and 1a was assessed by surface plasmon resonance (SPR). The in vitro binding Ka ratio with respect to the WT for each variant based on trastuzumab, as determined by SPR, is shown in Table 1. The ability of v1261 and AAC9, based on rituximab, to bind the different FcγRs was also assessed by SPR and is presented in Table 2. WT refers to wild type trastuzumab with neither CH2 nor any CH3 domain mutations.

The in vitro binding Ka ratio for the reference v791 and v1261 variants as determined by SPR (shown in Table 1 and Table 2, respectively) indicate that the change in affinities for the Fcγ receptors is not significantly impacted by the introduction of heterodimerizing mutations in either trastuzumab or rituximab. v791 and v1261 carry the heterodimerising CH3 domain mutations but none of the CH2 domain mutations. All variants with the AAC prefix developed here showed significantly decreased binding to all receptors. In most cases the binding was undetectable or unquantifiable due to the low affinity. The rituximab based AAC9 with the CH2 domain mutations also shows significantly reduced or undetectable binding to the Fcγ receptors. These observations are noteworthy in context of the detectable binding observed in the case of variant 1051 with the L234A/L235A double mutation, which is typically introduced as the mutation for avoiding Fcγ receptor binding.

### 2.3. Asymmetric Antibody Constructs Do Not Bind C1q

Since binding to the C1q complex is essential for activation of the complement pathway, the mutations were tested for their ability to knock-out C1q binding activity using SPR. Antibodies were indirectly captured onto HER2 bound to the SPR chip surface, and 30 nM C1q was injected over the antibody variants (see the Methods section). The C1q complex is an extremely large, multi-subunit protein that generates complex binding sensorgrams by SPR. Therefore binding is qualitatively assessed as reduction in the maximal binding signal compared to the wild-type trastuzumab variant. All of the variants showed undetectable binding to C1q, except for AAC1 which showed decreased, but detectable binding to C1q (Table 1, Supplementary Figure S1) relative to the SPR signal for wild-type reference antibody.

### 2.4. Asymmetric Antibody Constructs Bind to FcRn

A subset of the trastuzumab-based variants were tested for FcRn binding, and all of them showed similar binding compared to the WT control, indicating that the FcRn binding site was not compromised by the various mutations. FcRn binding was measured at both pH 6.0 and 7.4. Variants with WT binding at pH 6.0 and no detectable binding at pH 7.4 are denoted as “Yes” (Table 1).

### 2.5. Asymmetric Antibody Constructs Are Thermally Stable

**Figure 2:**
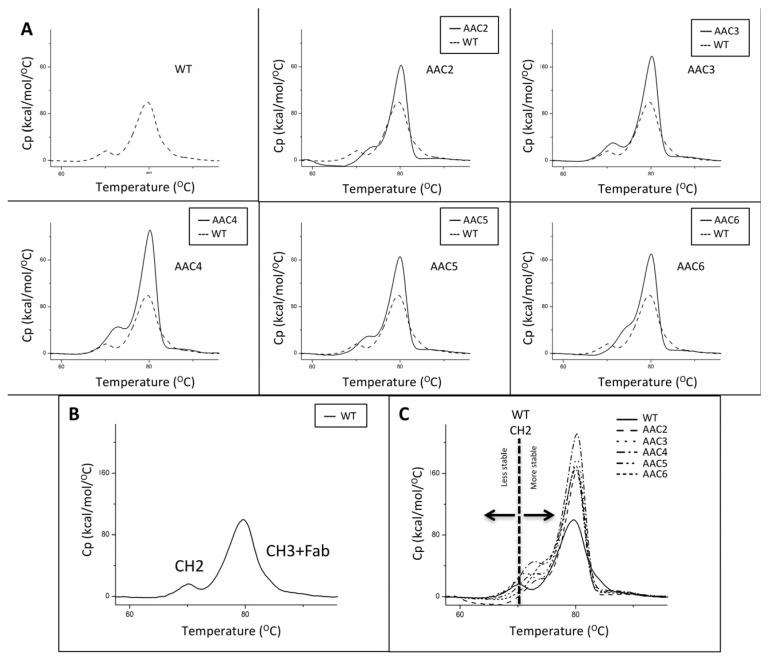
Thermograms of exemplary asymmetric antibody constructs compared to wild-type antibody (WT). (**A**) The thermograms for WT, and AAC2-AAC6. (**B**) The identity of the two transitions in the WT. The first transition corresponds to the unfolding of the CH2 domain, the second transition corresponds to the unfolding of the CH3 + Fab. (**C**) The overlay of a number of samples showing how the CH2 transition of the variants has shifted to a higher Tm value, indicating higher stability.

The thermal stability of the CH2 domains of the asymmetric antibody constructs was determined using differential scanning calorimetry. Thermal unfolding curves for the heterodimers tested were followed from 20 °C to 100 °C and are shown in Figure 2. The melting temperatures for the CH2 transition of the heteromultimers tested are shown in Table 1. These results show that a number of designs have higher Tm for the CH2 domain indicating greater stability in the CH2 domain when compared to the WT trastuzumab.

The thermal stability of v1261 and AAC9, constructs based on rituximab, was determined using differential scanning calorimetry. v1261 had a first transition Tm of 73 °C, which is the result of Fab + CH2. AAC9 had a Tm of 75 °C (not shown). The shift of +2 °C is consistent with what we observed with the equivalent trastuzumab-based AAC5 with the same CH2 mutations (Table 2, Figure 2).

### 2.6. IEX Analysis of Asymmetric Antibody

**Figure 3:**
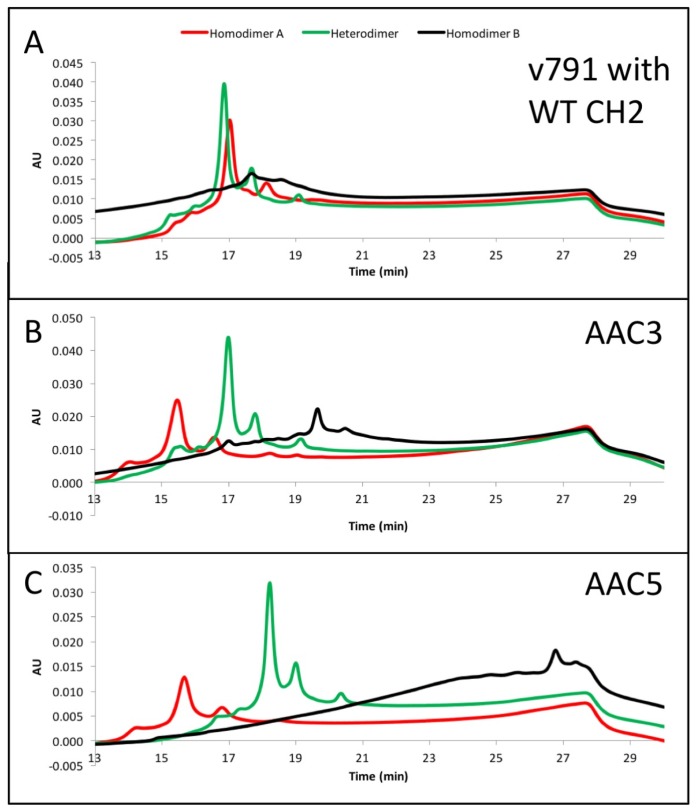

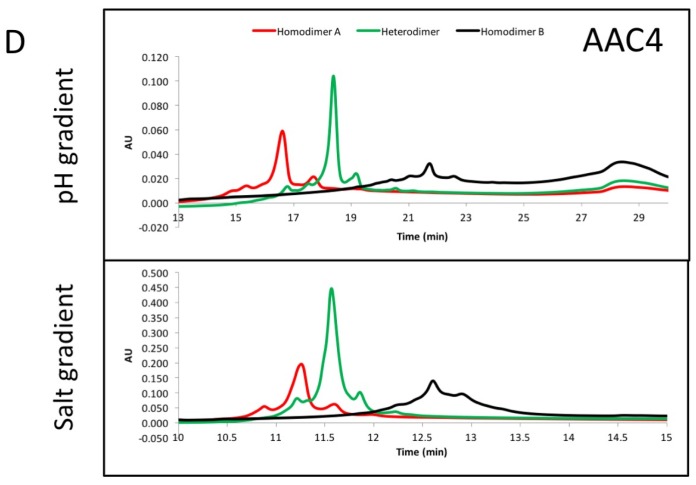
(**A**–**C**) exemplary results showing the resolution by ion exchange chromatography on an Agilent BioMAb (Weak Cation Exchange) column. The homodimers and heterodimers of asymmetric antibody constructs were independently expressed and purified. (**D**) separation of components using a pH gradient (low pH to high pH, upper panel), or a salt gradient (low salt to high salt, lower panel) using exemplary asymmetric variant AAC4.

The asymmetric charges introduced into the lower hinge region facilitated the identification and removal of homodimer contaminants. AAC3, AAC4 and AAC5 were expressed with ratios of chains A and B that would promote formation of homodimers of chain A (no chain B expressed, Figure 3, red), homodimers of chain B (no chain A expressed, Figure 3, black), or heterodimers (Figure 3, green). The resulting products were purified by protein A, followed by Size Exclusion Chromatography. This was necessary because expressing only chain A or chain B results in a large amount of half-antibodies and homodimers, and we pooled fractions that contained the largest amount of homodimers. The final products were analyzed by UPLC IEX (Ultra Performance Liquid Chromatography—Ion Exchange Chromatography) using a pH gradient for elution on a weak cation exchange column (Figure 3A–C). Traces for homodimer A, homodimer B or heterodimers, respectively are labelled. Figure 3A–C show that the separation of heterodimers and homodimer contaminants in variants into which asymmetric charges have been introduced in the lower hinge region is increased relative to the variant containing no CH2 mutations. All samples showed a major peak, followed by one or two smaller peaks corresponding to heterogeneity at the C-terminal lysine. Figure 3D demonstrates with variant AAC4 that similar resolution can be obtained using either pH gradient or salt gradient elution. These designs would facilitate process development for removal of homodimer contaminants. Combined with the mutations described above, introduction of asymmetric charges on the lower hinge region results in designs that decrease or eliminate receptor binding, impart greater thermal stability, and improve manufacturability by facilitating homodimer contaminant removal.

### 2.7. Asymmetric Antibody Construct 6 Based on Trastuzumab Do Not Stimulate ADCC (Antibody-Dependent Cell-Mediated Cytotoxicity) in SK-BR-3 Cells

**Figure 4:**
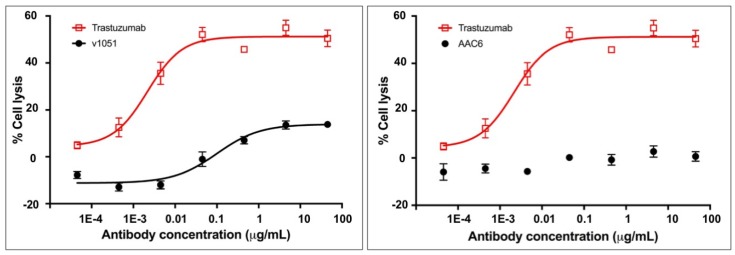
ADCC activity of a control variant (v1051) with the traditional symmetric L234A/L235A mutation for the knock-out of FcgR binding relative to an exemplary heteromultimer with asymmetric mutations in the CH2 domain (AAC6) against SK-BR-3 cells. An interpolated symmetrical sigmoidal shape is shown for Trastuzumab and v1051 (see Supplementary Table S1 for values derived from the fit). There was no convergence for AAC6. The error bars represent the standard error of the mean. For some points, the error bars would be shorter than the height of the symbol. In these cases, error bars are not drawn.

AAC6, an exemplary trastuzumab variant, was tested for its ability to stimulate ADCC in SK-BR-3 cells in order to assess whether the absence of measured binding to FcγR translated into an inability to mediate effector function as measured by ADCC. SK-BR-3 cells express HER2 on their surface and thus bind to trastuzumab, allowing for NK cell mediated ADCC in the presence of trastuzumab. The activity of AAC6 in this assay was compared to that of the control variant 1051 described in Table 1, and to the positive control trastuzumab. The results indicate that the exemplary heterodimeric AAC6 antibody silences ADCC activity in this assay (Figure 4, Supplementary Table S1). The effector function silencing with this variant is notably cleaner than that observed for variant 1051 with the L234A/L235A double mutation, thus corroborating the observation in the SPR binding assay.

### 2.8. Design from Asymmetric Antibody Construct 5 Is Selected as Lead

We had initially selected AAC6 as the lead variant. It had the highest thermal stability, no C1q or Fcγ receptor binding by SPR, and no ADCC activity. However, when we analyzed its IEX profile as we did for AAC3-5 in Section 2.6, it did not provide the added benefit of having a good separation of heterodimers and homodimers (Supplementary Figure S2).

On the other hand, the mutations in AAC5 allowed for excellent separation of homodimer impurities by IEX (Figure 3), and it also had higher than WT thermal stability, conserved FcRn binding, and no C1q or Fcγ receptor binding by SPR (Table 1). Hence the designed mutations in AAC5 were selected as new lead and introduced into an anti-CD20 antibody (AAC9) for measuring ADCC and CDC activities.

### 2.9. Asymmetric Antibody Construct Based on Rituximab Do Not Stimulate ADCC in Daudi Cells

**Figure 5:**
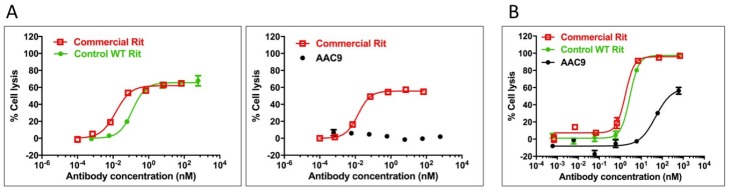
Ability of variants AAC9 to mediate ADCC (**A**) or CDC (**B**) against Daudi cells compared to controls including commercially available rituximab. An interpolated symmetrical sigmoidal shape is shown for Rituximab (see Supplementary Table S2 for values derived from the fit). There was no convergence for AAC9. The error bars represent the standard error of the mean. For some points, the error bars would be shorter than the height of the symbol. In these cases, error bars are not drawn.

AAC9, the rituximab-based variant, was tested for its ability to stimulate ADCC in Daudi cells in order to assess whether the lack of measured binding to FcγR translated into an inability to mediate effector function as measured by ADCC. Daudi cells express CD20 on their surface and thus bind to rituximab, allowing for NK cell mediated ADCC in the presence of rituximab. The activity of the variant in this assay was compared to that of the control rituximab variant described in Table 2, and to commercially obtained rituximab. The results are shown in Figure 5A and Supplementary Table S2 and indicate that AAC9 shows significantly decreased or undetectable ADCC activity.

### 2.10. Asymmetric Antibody Construct Based on Rituximab Reduce CDC (Complement-Dependent Cytotoxicity) in Daudi Cells

AAC9, the rituximab variant was tested to determine whether it was able to mediate CDC in Daudi cells. The activity of the Fc variants in this assay was compared to that of the control rituximab variant described in Table 2, and to commercially obtained rituximab. The results are shown in Figure 5B and Supplementary Table S2 and indicate that AAC9 shows significantly lower CDC activity.

## 3. Discussion

Therapeutic antibodies represent a major class of biopharmaceutical products, with a growing market presence [22]. One major area of research is the creation of bispecific antibodies, which can bridge different cell types, or convey higher specificity than natural antibodies [23]. Depending on the application of a bispecific antibody, it is sometimes necessary to engineer the molecule for effector function properties that differ from those naturally found in antibodies [6]. For example, it could be detrimental for a bispecific antibody that recognizes an immune cell to carry out ADCC, ADCP or CDC functions.

Until now, Fc engineering that changes the effector function of antibodies has been carried out with symmetric mutations in the CH2 domain. The recent creation of a number of different heterodimeric scaffolds opens up the possibility of engineering asymmetric mutations in the CH2 domain [18,19,20,24]. For example, Liu et al. and Mimoto et al. made use of asymmetric mutations to create an Fc variant with enhanced effector function [25,26]. Here, we report the first asymmetric designs with reduced effector function and increased thermal stability compared to wild-type CH2 domains (Table 1 and Table 2). The activity has been exemplified in two different antibody prototypes, trastuzumab and rituximab. The use of a heterodimeric heavy chain or common light chain antibodies as prototype is purely for the simplification of antibody preparation and characterization; the mutations described here are expected to perform equally well in the context of an actual bispecific antibody.

We tested mutations in loops of the CH2 domain, as well as in the lower hinge (Figure 1, Table 1 and Table 2). However, the main region we tested includes residues 233–234, which have been shown extensively to be important in mediating effector function [16,17,21]. The first design we tested was the well-known LALA (L234A/L235A) variant with mutations on both chains or just on one chain (v1051 and AAC1, Table 1). Measurement of binding to the high affinity FcγR1a receptor, as well as the FcγR3a receptor which is central for ADCC activity showed that mutations on both chains were necessary to minimize effector function. We also tested the effect of introducing both positively charged and negatively charged mutations at these positions, both of which reduced binding to the Fc gamma receptors. Importantly, the charged mutations increased Tm as measured by DSC (Figure 2). This is important, as other solutions for reduced effector function such as removal of the glycosylation site or point mutations in the Fc frequently result in decreases in thermal stability, and often need to be accompanied by additional mutations such as new disulfide bonds to bring thermostability back to WT levels [10], something not required in our reported designs.

The mechanism by which residues 234–235 mediate interactions with Fc gamma receptors was not well understood until very recently, as it is in a region that shows very distinct conformations in different structures, even of the same complex [27,28,29]. However, a number of Fc/FcγR1a structures reported in the last year show a clear interaction between L234–L235 with a hydrophobic pocket present in the FcγR1a receptor [30,31,32]. Hydrophobic pockets in the same region are present in FcγR2a and FcγR3a receptors, and although these leucine residues are not directly involved in the Fc/FcγR2a and Fc/FcγR3a complex structures as for Fc/FcγR1a, it is clear that the hydrophobicity of residues 234–235 is important in the recognition of the receptors. Hence, mutations to charged residues significantly decrease the interaction of the Fc with all of the measured receptors, and result in a clear decrease in ADCC and CDC activity (Table 1 and Table 2, Figure 5).

The CH3 heterodimeric design tested here consistently produces bispecific antibodies of high purity and high stability. Recently, Leaver-Fay et al. [23] produced new CH3 heterodimeric designs and compared the Tm of the CH3 domain for Genentech’s KH (69.9 °C) [18], Amgen’s charge inversion (68.8 °C) [19], their new designs (~71 °C) [24], and the CH3 mutations employed here (81.2 °C) [20]. DNA titrations may be required during transient expression of all CH3 heterodimerising designs to match expression of the two obligate heavy chains and minimize half-antibodies and homodimers [20]. For high throughput screening of variants produced by such transient expression, it would be ideal to have additional handles to purify the desired heterodimer. By introducing mutations of opposite charge in each chain such as in variant AAC5, we have also enhanced the ability of the heterodimeric Fc to be separated from its homodimeric impurities on the basis of charge differences. By introducing negative charges on chain A, we should lower its pI. Conversely, positive charges on chain B would increase its pI. We observe that the net result is a larger pI difference between the heterodimer and its homodimer impurities, making them amenable to separation by ion exchange chromatography, which has been shown to be a good technique to purify antibodies [33,34]. As shown in Figure 3A, the impurities were difficult to separate from the desired heterodimer containing WT CH2. However, asymmetric charged mutations significantly changed the elution profile of the impurities under the same elution gradient. AAC4 was tested with both a pH gradient and a salt gradient in a weak cation exchange column, and the current designs resulted in better separation of impurities using both methods. As predicted, homodimer A (with its lower pI) eluted first, then the heterodimer, and finally homodimer B (with its higher pI).

For many bispecific antibody applications, there is a need to produce molecules with abrogated FcγR and complement-mediated function, enhanced stability and potential to produce pure material based on additional handles that facilitate downstream purification. In the literature, there are several reports describing designs that excel in one or more of these requirements, but not all of them. From the results of AAC5 and AAC9, we have demonstrated that the use of the asymmetric mutations in the CH2 domain, L234D/L235E on one chain, and E233K/L234R/L235R on the other chain, is a viable strategy to produce bispecific antibodies that satisfy all of these requirements.

## 4. Materials and Methods

### 4.1. Expression and Purification of the Samples

#### 4.1.1. Antibody Constructs

The antibodies and controls were cloned and expressed as follows. Variants were prepared by site-directed mutagenesis using standard methods. The final DNA was sub-cloned into the vector pTT5 (see US Patent Publication No. US9353382 B2). Expression was carried out in either 2 mL or 50 mL or 500 mL CHO 3E7 cells. CHO cells were transfected in exponential growth phase (1.5 to 2 million cells/mL) with aqueous 1 mg/mL 25 kDa polyethylenimine (PEI^pro^, Polyplus Transfection SA, Illkirch, France) at a PEI:DNA ratio of 2.5:1 [35]. In order to determine the optimal concentration range for forming heterodimers, the DNA was transfected in optimal DNA ratios of the heavy chain A (HC-A), light chain (LC), and heavy chain B that allow for heterodimer formation (e.g., HC-A/HC-B/LC ratios = 25:25:50%). Transfected cells were harvested after 5–6 days with the culture medium collected after centrifugation at 4000 rpm and clarified using a 0.45 μm filter.

The clarified culture medium was loaded onto a MabSelect SuRe (GE Healthcare, Baie-d'Urfé, QC, Canada) Protein-A column and washed with 10 column volumes of PBS buffer at pH 7.2. The antibody was eluted with 10 column volumes of citrate buffer at pH 3.6 with the pooled fractions containing the antibody neutralized with TRIS at pH 11. The Protein-A purified antibody was further purified by size exclusion chromatography (SEC). For gel filtration, 3.5 mg of the antibody mixture was concentrated to 1.5 mL and loaded onto a Sephadex 200 HiLoad 16/600 200 pg column (GE Healthcare) equilibrated in PBS pH 7.4 via an ÄKTA Express FPLC at a flow-rate of 1 mL/min. Fractions corresponding to the purified antibody were collected, concentrated to ~1 mg/mL and stored at −80 °C

#### 4.1.2. FcγRs and FcRn

FcγR 2aH, 2aR, 2b, 3aF, and 3aV were produced in 293-6E cells while FcγR 1a (CD64) was produced in CHO-3E7 cells according to previous work [36]. The human FcRn was also expressed in 293-6E cells by the co-transfection of the alpha subunit (p51) extracellular domain containing a TEV-cleavable C-terminal his-tag with B2-microglobulin in a 1:1 ratio. Following purification as described in Dorion-Thibaudeau et al. [36], the C-terminal his-tag was removed by TEV cleavage.

### 4.2. Surface Plasmon Resonance

#### 4.2.1. FcγR Binding against Trastuzumab and Rituximab Constructs

Affinity of FcγRs to antibody Fc was measured by SPR using a ProteOn XPR36 at 25 °C with 10 mM HEPES, 150 mM NaCl, 3.4 mM EDTA, and 0.05% Tween 20 at pH 7.4 as the running buffer. For trastuzumab variants, recombinant HER-2 was immobilized on a GLM sensorchip using standard amine coupling with a BioRad amine coupling kit. Briefly, the GLM sensorchip was activated with NHS/EDC followed by injecting HER2 at 4.0 μg/mL in 10 mM NaOAc (pH 4.5) until approximately 3000 resonance units (RUs) were immobilized. This was followed by quenching the remaining active groups with ethanolamine. The same protocol was followed for the creation of the rituximab capture surface except for these variants a goat anti-IgG capture surface was created using 4.0 μg/mL goat-anti-human Fc (Jackson Immunoresearch) in 10 mM NaOAc (pH 4.5). A blank goat-anti-human-Fc surface was always left open to reference any non-specific binding in the interaction. Wild-type trastuzumab or rituximab variants were indirectly captured onto their SPR surface by injecting a 40 μg/mL solution purified antibody in the ligand direction at 25 μL/min for 240 s resulting in approx. 500 RUs on the surface. Following buffer injections to establish a stable baseline in the analyte direction, analyte was injected at 50 μL/min for 120 s with a 180 s dissociation phase to obtain a set of binding sensorgrams. Five concentrations of a 3-fold dilution series of the FcγRs with 10 μM top nominal concentrations for all receptors were used except 30 nM for FcγR1a, and buffer was included for double referencing. Resultant Kd (affinity) values were determined from the aligned and referenced sensorgrams using the Equilibrium Fit model in Proteon Manager v3.1.0 with reported values as the mean of two or three independent runs.

#### 4.2.2. C1q

The ability of the asymmetric antibody constructs to bind to C1q was tested as follows. Human C1q was purchased from GenWay Biotech (San Diego, CA, USA). 30 nM of C1q were injected using top-nominal 3-fold dilutions (30/10/3.33/1.11/0.37 nM) over mAb variants captured onto the same HER2 SPR surface used for FcγR binding determination using the protocols described in Section 4.2.1. Due to the avidity of the hexameric C1q, only qualitative data was obtained, where the binding was classified as observed (yes), diminished compared to the WT control (partial) or not detected (NB) as summarized in Table 1 and shown in Supplementary Figure S1.

#### 4.2.3. FcRn

The ability of the asymmetric antibodies to bind to FcRn was tested by SPR as follows. SPR chip capture surface was prepared with goat anti-hIgG polyclonal as in Section 4.2.1. Variants were captured from supernatants in the ligand direction. FcRn was injected at 1 μM maximum with a 3× dilution series at 50 μL/min for 120 s in the analyte direction. Duplicate runs in 10 mM MES pH 6 were referenced to a blank capture surface and qualitatively produced similar results. Due to the complexity of the sensorgrams, relative binding to the controls only was assessed. One run at pH 7.4 was performed to check for lack of binding. The results are shown in Table 1.

### 4.3. Differential Scanning Calorimetry

Each antibody construct was diluted to 0.2 mg/mL in PBS, and a total of 400 μL was used for DSC analysis with a VP-Capillary DSC (GE Healthcare). At the start of each DSC run, five buffer blank injections were performed to stabilize the baseline, and a buffer injection was placed before each antibody injection for referencing. Each sample was scanned from 20 to 100 °C at a 60 °C/h rate, with low feedback, 8 s filter, 5 min preTstat, and 70 psi nitrogen pressure. The resulting thermograms were referenced and analyzed using Origin 7 software.

### 4.4. UPLC IEX

Chain A and Chain B of variants 791 (WT heterodimer), AAC3 (L234D/L235E[Chain A]|L234K/L235K[Chain B]) and AAC5 (L234D/L235E[Chain A]|E233K/L234K/L235K[Chain B]) were expressed in ratios 1:0 (A), 1:1 (C) and 0:1 (E) in 50 mL CHO cultures. Ratios A and E produced homodimers of Chain A and Chain B, respectively. All of the samples were purified by Protein A, and then by Size Exclusion Chromatography (SEC) using a Superdex 200 16/600 column in PBS buffer prior to loading them into the UPLC IEX. UPLC IEX was carried out under the following conditions (pH gradient): Solvents: A, 0.1 M NaH_2_PO_4_, pH 4.44; B, 0.1 M Na_2_HPO_4_, pH 9.20; C, MilliQ water; D, 0.5 M NaOAc, pH 9.13 (lot#03-Dec-12). Initial Buffer: 18% A, 2% B, 68% C, 12% D = 20 mM NaPO_4_, 60 mM NaOAc, pH ~5.9; Gradient: to 2% A, 18% B, 68% C, 12% D = 20 mM NaPO_4_, 60 mM NaAcetate, pH ~7.9 in 7.2 column volumes. Flow rate: 0.3 mL/min. Temperature: 30 °C. Pressure: ~4200 psi. Column: Agilent BioMAb (Weak Cation Exchange), 4.6 × 50 mm, 1.7 μm particles, SN USDJA01061. For AAC4, it was eluted under a pH gradient as described above, or under a salt gradient as follows: Solvents: A 0.1 M NaH_2_PO4, pH 4.44; B 0.1 M Na2HPO4, pH 9.20; C MilliQ water; D 0.5 M NaCl. Initial Buffer: 18% A, 2% B, 68% C, 12% D = 20 mM NaPO4, 60 mM NaCl, pH ~5.9. Salt gradient to 18% A, 2% B, 0% C, 80% D (=20 mM NaPO_4_, 400 mM NaCl, pH ~5.9) in 7.2 column volumes.

### 4.5. ADCC (Antibody-Dependent Cell-Mediated Cytotoxicity)

The target cell lines used were a Daudi cell line (ATCC, Cat# CCL-213), or SK-BR-3 (ATCC#HTB-30), with NK92/CD16a(158V/V) cells used as effectors. Rituximab and Trastuzumab were used as positive control for the Daudi and SK-BR-3 cell lines, respectively. Frozen cells were thawed by gently swirling the vial in the 37 °C water bath. The cell suspension was then transferred to a 15 mL centrifuge tube, followed by addition of 5 mL of pre-warmed complete medium. After centrifugation for 3–5 min at 500 g, the supernatant was aspirated. 10 mL of complete medium was added and the cells were resuspended by pipetting up and down for a few times. Cell viability was determined by Trypan Blue staining method. The cell suspension was then seeded in flasks. The cells were incubated at 37 °C, 5% CO_2_ overnight. Cells were maintained at 37 °C/5% CO_2_ and regularly sub-cultured with suitable medium supplemented with 10% FBS according to protocol from ATCC. The antibody sample and the standard were delivered in dry shipper and stored at −20 °C before testing. The sample and the standard were stored at 4 °C after they were thawed on ice. The sample and the standard were diluted with Phenol red free MEM medium (supplemented with 1% FBS and 1% Pen/Strep) and applied to the tests. ADCC assay buffer was composed of 98% Phenol red free MEM medium, 1% Pen/Strep and 1% FBS. NK92/FcγR3a (158V/V) cells were conventionally maintained. Target cells were harvested by centrifugation at 800 rpm for 3 min, washed with assay medium once and centrifuged; the medium above the pellet was removed completely. Cells were gently suspended with assay medium to make a single-cell solution. The target cell number was adjusted to 4× cell stock (10,000 cells in 50 μL assay medium). Test articles were prepared at indicated concentrations. 50 μL 4× target cell stock were seeded to 96-well assay plates and 50 μL 4× sample diluents added. The plates were incubated at room temperature for 30 min in cell culture incubator. 100 μL effector cells (E/T = 5:1, i.e., 50,000 effector cells per well) were added to initiate the reaction and mixed gently by cross shaking. Triton X-100 was added to cell controls without effector cells and antibody in a final concentration of 1% to lyse the target cells and it served as the maximum lysis control; assay buffers were added to cell controls without effector cells and antibody and it served as the minimum LDH release control. Target cells incubated with effector cells without the presence of antibodies were set as background control of non-specific LDH release when both cells were incubated together. Plates were incubated at 37 °C/5% CO_2_ incubator for 4–6 h. The cell viability was assayed with an LDH kit. The absorbance data at OD492 nm and OD650 nm were measured on Flexstation 3. The background (OD650 nm) subtracted OD492 nm data was analyzed to study the LDH release. The percentages of cell lysis were calculated according to the formula:

% Cell lysis = 100 × (1 − (OD_Sample data_ − OD_tumor cells plus effector cells_)/(OD_Maximum release_ − OD_Minimum release_))

### 4.6. Asymmetric Antibody Constructs Based on Rituximab Reduce CDC (Complement-Dependent Cytotoxicity) in Daudi Cells

A Daudi cell line (ATCC, Cat# CCL-213), NK92/CD16a (158V/V) was used. Rituximab (Rituxan) was used as the positive control antibody. Daudi cells were harvested by centrifugation and the pellets were washed with assay buffer once. Viable cells were counted by Trypan Blue dye. Cell population was only allowed of >99% viability for the assay. The cell concentration was adjusted and 5000 cells were seeded in 20 μL CDC buffer. 10 μL diluted samples were added (8 concentrations with a dilution factor of 1:10 descending from 600 nM, in triplicates). Samples and rituximab control were incubated at room temperature for 30 min. 10 μL normal human serum (NHS), which constituted 10% final concentration in 40 μL reaction volume, were added to each well to initiate the CDC assay. Plate was incubated at 37 °C/5% CO_2_ incubator for 2 h. Cell viability tests were performed with CellTiter-Glo^®^ Luminescent Cell Viability Assay Kit (Promega, Cat# G7571). Luminescence was read on PHERAStar Plus (BMG Labtech) and record the relative light unit (RLU) data. The percentage of cell lysis was calculated with the formula:

% Cell lysis = 100 × (1 − (RLU_sample_)/(RLU_cell+NHS_)

## Figures and Tables

**Table 1:** CH2 mutations, transient expression yield, FcγR/C1q/FcRn binding and thermal stability of Asymmetric Antibody Constructs (AAC).

	CH2 Mutations ^1^		Yield ^2^ [mg/L]	SPR Ka_mut_/Ka_WT_^4^						SPR Binding C1q ^5^	SPR Binding FcRn ^6^	DSC Tm (CH2 Transition) ^7^ [°C]
Variant	HC-A	HC-B		2aH	2aR	2b	3aF	3aV	1a			
WT ^8^	-	-	30	30	1	1	1	1	1	Yes	Yes	71
791 ^9^	-	-	73 ^10^	1.7	1.7	1.2	n/d ^3^	1.3	n/d	Yes	n/d	72
1051 (control)	L234A/L235A	L234A/L235A	48	0.06	0.06	0.52	0.29	0.10	0.01	NB	Yes	72
AAC1	L234A/L235A	-	n/d	n/d	n/d	n/d	0.87	0.71	0.48	partial	n/d	n/d
AAC2	L234A/L235A	L234K/L235K	63	NB	NB	NB	LOW	LOW	LOW	NB	Yes	74
AAC3	L234D/L235E	L234K/L235K	39	NB	NB	NB	LOW	LOW	LOW	NB	Yes	72
AAC4	E233A/L234D/L235E	E233A/L234R/L235R	42	NB	NB	NB	LOW	LOW	LOW	NB	Yes	73
AAC5	L234D/L235E	E233K/L234R/L235R	44	NB	NB	NB	LOW	LOW	LOW	NB	Yes	73
AAC6	E233A/L234K/L235A	E233K/L234A/L235K	31	NB	NB	NB	NB	LOW	LOW	NB	Yes	75
AAC7	E269Q/D270N	E269K/D270R	n/d	n/d	n/d	n/d	LOW	LOW	0.15	NB	n/d	n/d
AAC8	-	L235K/A327K	n/d	n/d	n/d	n/d	0.19	0.10	0.13	NB	n/d	n/d

^1^ All sequences described herein are numbered using the EU numbering system. In addition to the mutations shown in the table, all variants, except the WT, had the following modifications in the heavy chain CH3 domain introduced in order to promote the formation of a heterodimer Fc domain. Chain A: T350V/L351Y/S400E/F405A/Y407V. Chain B: T350V/T366L/N390R/K392M/T394W. ^2^ Yield after protein A purification for some of the variants from a 50 mL expression. ^3^ n/d = not determined. ^4^ Ka_mut_/Ka_WT_ = Kd_WT_/Kd_mut_; Receptors were injected at a high-concentration of 10.0 μM (FcγR2aR, FcγR2b), 6.0 uM (FcγR3aF, FcγR3aV), or 30 nM (FcγR1a), each in 3-fold dilution series. LOW means that binding was detected by a small shift in the sensorgrams but the binding was too weak to quantify, NB means no binding was detected under the conditions tested. The Kd of the WT against the 2aH receptor (CD32aH) was 0.48 μM. The Kd of the WT against the 2aR receptor (CD32aR) was 0.87 μM. The Kd of the WT against the 2b receptor (CD32b) was 3.4 μM. The Kd of the WT against the 3aF receptor (CD16aF) was 1.9 μM. The Kd of the WT against the 3aV receptor (CD16aV) was 0.60 μM. The Kd of the WT against the 1a receptor (CD64a) was 0.65 nM. ^5^ C1q is a hexamer of heterotrimers with a potential stoichiometry mAb:C1q of 6:1. The binding kinetics were very complex, and a proper Kd could not be determined. Receptor was tested at 30 nM. ‘partial’ means diminished binding, ‘NB’ means no detectable binding. Sensograms are shown in Supplementary Figure S1. ^6^ FcRn binding was measured at pH 6.0 and 7.4. Variants with WT binding at pH 6.5 and no detectable binding at pH 7.4 are denoted as ‘Yes’. ^7^ The Tm was measured by deconvolution using a non-2 state model of the first transition in the thermograms shown in Figure 2. ^8^ Homodimeric trastuzumab with no mutations was used as WT. ^9^ Variant 791 contains only CH3 mutations, and no CH2 mutations. ^10^ Variant 791 was expressed in a different batch, where it gave a yield of 73 mg/L, and homodimeric trastuzumab gave a yield of 62 mg/L.

**Table 2:** FcγR binding, thermal stability and ADCC/CDC activity of the lead Asymmetric Antibody Construct as an anti-CD20 Rituximab variant.

	CH2 mutations ^1^		SPR Kamut/KaWT ^2^					DSC Tm ^3^ (CH2 Transition) (°C)	ADCC		CDC	
Variant	Chain A	Chain B	2aH	2aR	2b	3aF	3aV		EC50 (nM)	Maximum Lysis	EC50 (nM)	Maximum Lysis (%)
Control Rituximab v1261	-	-	1.9	1.5	1.9	1.4	1.34	73	0.1	66	2.9	96
AAC9	L234D/L235E	E233K/L234R/L235R	NB	NB	NB	NB	0.08	75	Non-lytic	Non-lytic	>10 *	<60 *

^1^ All sequences described herein are numbered using the EU numbering system. In addition to the mutations shown in the table, all variants including control rituximab had the following modifications in the heavy chain CH3 domain introduced in order to promote the formation of a heterodimer Fc domain. Chain A: T350V/L351Y/F405A/Y407V, and Chain B: T350V/T366L/K392L/T394W. ^2^ Homodimeric trastuzumab with no mutations was used as WT reference. Ka_mut_/Ka_WT_ = Kd_WT_/Kd_mut_; Receptors were injected at a high-concentration of 10.0 μM (FcγR2aR, FcγR2b), 6.0 μM (FcγR3aF, FcγR3aV), or 30 nM (FcγR1a), each in 3-fold dilution series. LOW means that binding was detected by a small shift in the sensorgrams but the binding was too weak to quantify, NB means no binding was detected under the conditions tested. The Kd of the WT against the 2aH receptor (CD32aH) was 0.48 μM. ^2^ The Kd of the WT against the 2aR receptor (CD32aR) was 0.87 μM. The Kd of the WT against the 2b receptor (CD32b) was 3.4 μM. The Kd of the WT against the 3aF receptor (CD16aF) was 1.9 μM. The Kd of the WT against the 3aV receptor (CD16aV) was 0.60 μM. The Kd of the WT against the 1a receptor (CD64a) was 0.65 nM. ^3^ The first transition included the unfolding of both the rituximab FAB and CH2 domain. The Tm was measured by deconvolution using a non-2 state model of the first transition. * Lysis was reduced, but plateau was not reached and maximum lysis was not determined (Figure 5B). EC50 values appear to be greater than 10 nM, and lysis was <60% at the highest tested concentration.

## Supplementary Materials

The following are available online at www.mdpi.com/2073-4468/6/2/7/s1.
