# Supplementary material for: Asymmetric Fc Engineering for Bispecific Antibodies with Reduced Effector Function
Source: Antibodies (Basel). 2017 May 16;6(2):7. doi: 10.3390/antib6020007 (PMC6698841; doi:10.3390/antib6020007)
Supplement: Supplementary file 1 [file antibodies-06-00007-s001.pdf]

Supplementary Figure S1

Sensograms for the asymmetric antibody constructs binding to C1q (see methods). Due to the avidity of the hexameric C1q, only qualitative data was obtained, where the binding was classified as observed (yes, e.g. WT), diminished compared to the WT control (partial, e.g. AAC1) or not detected (NB) as summarized in Table 1. The different traces correspond to 30 nM top-nominal 3-fold dilutions: 30 nM (red), 10 nM (cyan), 3.33nM (blue), 1.11nM (green), 0.37 nM (magenta). v791* had C-terminal His and mRFP tags.

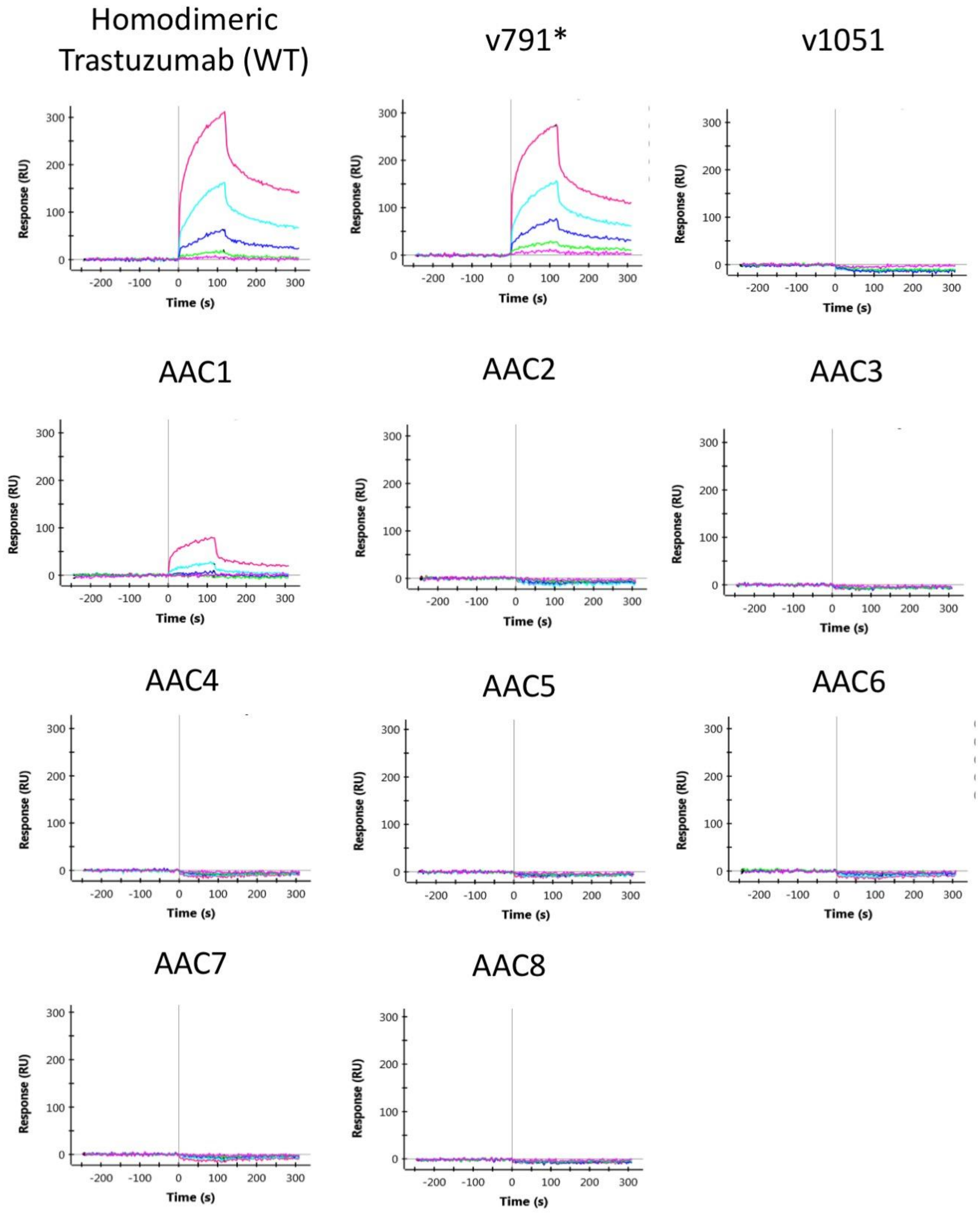

Supplementary Figure S2

Exemplary result showing the resolution by ion exchange chromatography of homodimers and heterodimers of asymmetric antibody constructs independently expressed and purified for v791 (A) and AAC6 (B) using a pH gradient (from low to high) on a weak cation exchange column.

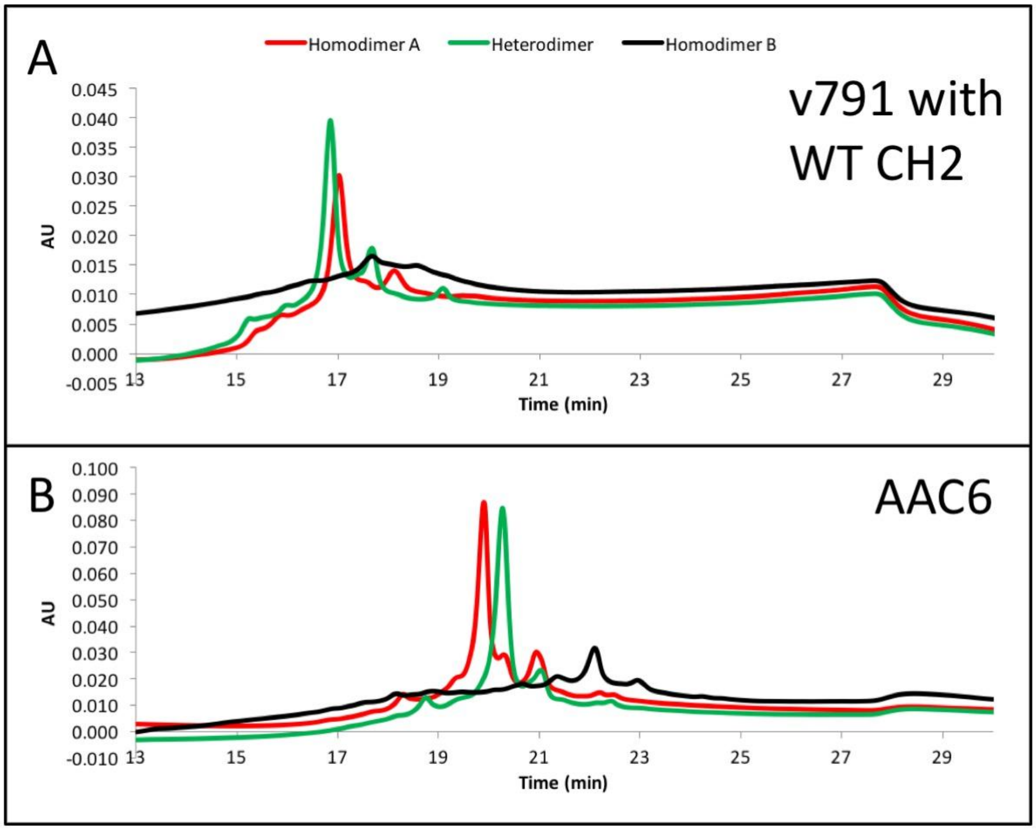

Supplementary Table S1 – Derived parameters from the interpolated symmetrical sigmoidal shape shown in Figure 4. The standard equation Y=Bottom + (Top-Bottom)/(1+10^((LogEC50-X)*HillSlope)) was used using the software Prism.

**Parameter**	**Trastuzumab**	**v1051**	**AAC6**
Span (Top-Bottom)	47%	25%	Poor fit
EC50 (μg/mL)	2.2E-3	9.9E-2	
HillSlope	1.1	0.84	
R^2	0.95	0.91	

Supplementary Table S2 – Derived parameters from the interpolated symmetrical sigmoidal shape shown in Figure 5. The standard equation Y=Bottom + (Top-Bottom)/(1+10^((LogEC50-X)*HillSlope)) was used using the software Prism.

ADCC

**Parameter**	**Commercial Rit**	**Control WT Rit**	**AAC9**
Span (Top-Bottom)	62	65	Poor fit
EC50 (nM)	1.4E-2	1.2E-1	
HillSlope	1.0	1.2	
R^2	0.98	0.99	

CDC

**Parameter**	**Commercial Rit**	**Control WT Rit**	**AAC9**
Span (Top-Bottom)	88.6	96.3	Plateau not reached
EC50 (nM)	1.8	2.9	
HillSlope	2.1	2.1	
R^2	0.98	0.99	
